# Exploring primary health care nurses’ perceptions of cervical cancer screening in Leribe, Lesotho

**DOI:** 10.4102/phcfm.v17i1.4942

**Published:** 2025-09-05

**Authors:** Maliketso G. Polane, Siyabonga B. Dlamini

**Affiliations:** 1 Department of Public Health Medicine, College of Health Sciences, University of KwaZulu-Natal, Durban, South Africa; 2 Cancer and Infectious Diseases Epidemiology Research Unit, College of Health Sciences, University of KwaZulu-Natal, Durban, South Africa

**Keywords:** cervical cancer screening, nurses’ perceptions, negative perceptions, positive perceptions, subsequent screening

## Abstract

**Background:**

Cervical cancer ranks fourth among cancers recorded globally and is the second most common cause of cancer-related morbidity and mortality in women. Although cervical cancer is fatal, the early discovery of precancerous cells by extensive and recurrent screening could lead to a significant decline in incidence. However, the acceptance of cervical cancer screening is low, even among healthcare workers.

**Aim:**

To explore the perceptions of primary care nurses about cervical cancer screening.

**Setting:**

The study was carried out in four primary health care centres (PHCCs) in the Leribe district.

**Methods:**

This is an exploratory qualitative study. The researcher purposively selected and interviewed 10 nurses at the selected PHCCs. The data were analysed thematically.

**Results:**

Nurses’ perceptions of cervical cancer screening influenced whether they routinely detect the disease. Certain perceptions, such as being susceptible to cancer, fear of cancer consequences, feeling relieved by negative results, high self-efficacy, training and witnessing deaths, all encouraged routine screening. Those that discouraged routine screening included fear of positive testing, lack of results, perceived lack of confidence and privacy in screeners and low self-efficacy.

**Conclusion:**

These findings show that nurses’ decisions to undergo a regular screening are either encouraged or discouraged by their perceptions about cervical cancer screening. They also imply that the general public may be impacted by these perceptions as well.

**Contribution:**

These findings add significantly to the body of knowledge about how policies can be improved to improve nursing screening programmes, which can improve screening rates among the general population.

## Introduction

An estimated 604 127 cases of cervical cancer and 341 831 deaths were recorded worldwide in 2020.^1^ Singh et al.^1^ further explained that in countries with low Human Development Index (HDI), cervical cancer incidence was three times higher than in countries with very high HDI, and similarly the mortality rate was six times higher in countries with low HDI compared to countries with very high HDI. Bray et al.^2^ further explained that cervical cancer ranks fourth in terms of incidence and mortality among women, with new estimated cases at 660 000 and 350 000 deaths recorded worldwide in 2022. However, the acceptance of cervical cancer screening is low, even among healthcare workers (HCWs).^3^ More than 80 000 African women receive a cervical cancer diagnosis yearly, with a 75% mortality rate.^4^ The majority are found in sub-Saharan Africa (SSA), where the incidence is relatively high, with an incidence rate of 50 per 100 000 women.^4^ In Lesotho, cervical cancer is the most common cancer, with an incidence of 29.5% of all cancer diagnoses, followed by breast cancer, with an incidence of 9.2% in the country in 2022.^5^ As a result, high rates of morbidity and mortality from cervical cancer can have an impact on the economy of any country, the psychosocial well-being and the overall quality of life of those who are impacted.^6^

Although cervical cancer is fatal, Mkhonta and Shirinde^7^ stated that it has a high survival rate when detected and treated early. Therefore, there has been a significant decline in the incidence of cervical cancer in western countries because of the introduction of screening techniques.^8,9^ Anyebe et al.^3^ agree that the early discovery of precancerous or early cancer lesions by extensive and recurrent screening using the following methods is responsible for the significant decrease in incidence. These techniques include Pap smear, visual inspection with acetic acid (VIA), visual inspection with Lugol iodine (VILI), colposcopy and human-papillomavirus deoxyribonucleic acid (HPV DNA) testing.^8^

Lesotho has adopted some of these measures and introduced a free cervical screening programme employing the VIA, VILI and Pap smear to reduce the incidence of cervical cancer.^10^ Despite these initiatives, uptake remains low.^10^ The two Lesotho studies conducted among all population proved the low cervical cancer screening uptake. The study done by Mphunyane and Nyangu^11^ showed that 48.0% of participants did not undergo screening. Another one conducted by Harder et al.^12^ found that 40.5% of respondents reported never having undergone screening. The low uptake could possibly be because of the perception of pain and discomfort, the lack of healthcare facilities and the feeling that it is not necessary to get tested as cited by the participants in the study conducted by Owolabi and Adejumo.^13^ As nurses play a significant role in educating the public about the availability and necessity of cervical cancer screening services, they are constantly viewed as role models in health-related issues.^13,14^ Some studies further explained that despite the high level of knowledge about cervical cancer screening and its advantages, nurses in some contexts have reportedly adopted and used cervical cancer screening services at relatively low rates.^13,15^ Tay et al.^16^ conducted a study in Singapore, which showed that out of the 815 nurses who reported on their screening history, 368 reported not having undergone screening. In another study conducted in Nigeria by Ifemelumma et al.,^17^ the results showed that screening uptake among nurses was low, as only 20.6% of the participants had ever taken a screening test. This poses an even more significant threat to the healthcare delivery system, as Oyekale et al.^18^ indicated that nurses comprise the majority of healthcare professionals and are essential resources for any country. Therefore, maintaining their health and safety is vital in providing ongoing patient care. However, previous studies done in Lesotho have focused on knowledge and attitudes towards cervical cancer screening in the general population.^10,19^ Little has been done about cervical cancer screening among nurses, let alone their perceptions about cervical cancer screening. Therefore, this study aimed to understand the perceptions of nurses about cervical cancer screening because Suantika et al.^20^ explained that nurses are less likely to participate in the early detection of cervical cancer. This is because of nurses’ perceptions that there is no risk factor for them to develop cervical cancer. Since nurses have never experienced symptoms, they are not sure of its prevention results or effectiveness and do not perceive the benefits of early detection.^20^

## Research methods and design

### Study design

This was a qualitative exploratory investigation in which an inductive approach was used. Armat et al.^21^ explain that at the beginning of a study, the mind of a researcher who uses an inductive approach is not entirely blank; rather, they have research question(s) and aims that direct their analysis. Moreover, according to Mbaka and Isiramen,^22^ exploratory and qualitative research is used to gain a detailed understanding of human behaviour, intentions, experience, motivations and attitudes based on observations and interpretations to find people’s thoughts and feelings. Mbaka and Isiramen^22^ further explained that exploratory and qualitative research is conducted when a problem has not been precisely defined and little is known about the phenomenon. Therefore, we conducted this type of study because, in Lesotho, cervical cancer screening has received a lot of attention among the general public, but little is known about screening among nurses, not to mention their perceptions about screening.

### Setting

The Leribe district, where this study was conducted, is the second most populated district in Lesotho, with a population of 298 352.^23^ In Lesotho, the healthcare system is made up of primary, secondary and tertiary facilities.^24^ The primary facilities in which this study took place are described by Mostafa et al.^24^ as the first point of contact for patients within the healthcare system, offering preventive and curative services. There are 25 primary health care centres (PHCCs) in Leribe; 4 centres were selected. All the centres are nurse driven, and they all offer cervical cancer screening services. According to the Lesotho Guidelines for the Screening and Treatment of Cervical Pre-Cancer, patients identified in these health centres as suspected cases of cervical cancer are referred to the hospital for treatment, including biopsy and staging.

### Study population

#### Inclusion criteria

The unit of analysis in this study was the primary health care (PHC) nurses, of all age groups and all levels of work experience, working in the PHCCs in Leribe, who had undergone screening for cervical cancer.

#### Exclusion criteria

Male nurses and women nurses who had never undergone cervical cancer screening were excluded.

### Sampling strategy

Two to three participants were selected for the four identified facilities. The ages of the nurses ranged from 29 years to 62 years. The researcher conducted interviews in close-by PHCCs and then moved to distal PHCCs. Therefore, this study employed a purpose sampling technique, particularly the maximum variation sample, in which participants with various work experiences, training, skills and different age groups were selected.

### Data collection

To invite women nurses willing to participate in this study, the study’s information sheet, which included invitations to participate, was sent to the District Health Management Team (DHMT) to distribute or send to all women nurses within the district. The researcher made telephone appointments with the facility managers about the interviews. On the scheduled interview days, the researcher interviewed nurses who were eligible, available and willing to participate. According to Sim et al.,^25^ the sample size in qualitative research is determined by saturation. Saturation is explained by Sarfo et al.^26^ as a data adequacy point where no more information could be gained from the respondents. In this study, saturation was reached at the tenth interview.

In-depth face-to-face, semi-structured interviews, which lasted for 20 to 30 min, were conducted using an interview guide (Online Appendix 1) with a reliable audio recorder (participants granted permission for recording), enabling the researcher to observe non-verbal behaviour. The semi-structured interview guide allowed the researcher to ask spontaneous questions, follow-up questions and seek more information. The researcher did not take any field notes as she was observing the participants for non-verbal behaviour. Although the interviews were conducted in English, the participants were allowed to respond in Sesotho, their home language, allowing them to express themselves in depth.

### Data analysis

Following the in-depth face-to-face recorded interviews, the researcher translated and transcribed the audiotapes from Sesotho into English. The transcribed data were then analysed using thematic analysis, using the following steps as explained by Maguire and Delahunt.^27^ The first step, becoming familiar with the data: at this step, the researcher began by listening to, translating and transcribing the recorded interview transcripts repeatedly. Once the data were compiled and organised, the second step, generating the initial codes, began with coding the chunks of data, where the initial codes were put into meaningful categories. This second step led to the third step, searching for themes in which the potential themes were discovered. In the fourth step, the discovered themes were reviewed and defined. The themes were then reviewed and refined based on the relevant and supporting data from the transcripts. After the themes were finalised, in the fourth step, they were further divided into sub-themes. Finally, in the last step, the data put together as themes and sub-themes had to be assembled to produce analytical results, which were then translated into interpretations.

### Measure of trustworthiness

Ahmed^28^ explains that because of the subjective nature of qualitative findings, it is vital to ensure trustworthiness in establishing the reliability and credibility of qualitative findings. Haq et al.^29^ further explain that credibility, transferability, dependability and confirmability of research design, process and action, can all be used to measure how trustworthy a qualitative study is.

Credibility is described as the extent to which the findings correctly reflect the truth about what the participants went through.^28^ To ensure credibility, member checking was used. Member checking was done by going back to some of the participants after data were analysed and interpreted so that the participants could confirm whether they were correctly interpreted. To maintain the study’s credibility, exact excerpts from the transcripts were utilised while presenting the findings.

Transferability refers to the extent to which qualitative research findings can be transferred to other settings with different respondents.^28^ Transferability was ensured through the provision of a thick description, that is sufficient details about the setting, sample characteristics, data collection and analysis methods. The study’s use of purposive sampling, which would also allow other researchers to concentrate on reliable sources who are especially knowledgeable about the topics under investigation, further guaranteed transferability.

Dependability refers to the consistency of results over comparable contexts.^29^ According to Haq et al.,^29^ if a study’s findings can be repeated in a setting or population with similar characteristics, then the study’s findings are considered dependable. To ensure a study’s dependability, Haq et al.^29^ assert that expert opinion is highly beneficial in enhancing data collection methods, processes, analysis plans and study interpretation. Therefore, from the beginning until the end of this project, the principal investigator, who has training in qualitative research, was guided by an experienced supervisor to ensure dependability.

Confirmability aims to ensure that the study’s findings are not impacted by any biases or preferences of the researcher.^28^ Confirmability in this study was ensured using a reflexive journal in which the researcher’s preconceived ideas about the research topic were noted so that they did not interfere with the interpretations of the findings.

### Reflexivity

According to Palaganas et al.,^30^ reflexivity is an ongoing process in which the researchers introspect on their values and awareness of evaluating and understanding how their social backgrounds, locations and assumptions can affect their research. When conducting this research, the researcher engaged in reflexivity by providing her autobiography. The researcher was born and bred in Lesotho, the Berea district. She attended primary school and high school in Berea and then trained to be a nurse at Maluti Adventist College, where she graduated with a diploma in General Nursing and Midwifery in 2009. She started working the same year at a PHCC in Leribe named Maputsoe Seventh Day Adventist Health Centre. During the years of her practice as a PHC nurse in that facility, cervical cancer screening was done through Pap smear, mainly in hospitals. As time went on, the visual inspection methods were introduced, and PHC nurses were capacitated in conducting screening at the PHCC level.

At the time when the research was conducted, the researcher was working in another PHCC named Emmanuel Health Centre. This time around, cervical cancer screening was mostly done at PHCCs through mainly visual inspection methods and a few smears. The researcher became interested in this topic because she needed to get an in-depth understanding of PHC nurses’ perceptions of cervical cancer screening.

The preceding autobiography was used to neutralise the impact of the researcher’s subjectivity. The neutralising work was achieved through bracketing, in which a researcher kept a reflexive journal to reflect on her perceptions.

### Ethical considerations

Ethical approval to conduct this study was obtained from the University of KwaZulu-Natal Biomedical Research Ethics Committee (No. BREC/00006211/2023). It was ensured that participation was voluntary and that participants had the right to withdraw from the study at any time. Before participating in the study, participants signed an informed consent form and granted verbal consent for recording. Participant codes were used to reference verbatim quotes.

## Results

The findings of the study (see Table 2) are presented under the broad themes identified from the data that addressed the objective of the study, which was to describe the perceptions of PHC nurses about cervical cancer screening in the Leribe district in Lesotho. From the analysis of the findings, six themes (perceived susceptibility, fear of perceived disease severity, outlook on the perceived benefits of cervical cancer screening, perceived barriers to cervical cancer screening, self-efficacy in cervical cancer screening and cues to action) and sub-themes (multiparity, being sexually active, cancer consequences, feelings of relief, early disease detection, fear of testing positive, lack of results, perceived lack of privacy and confidentiality, high self-efficacy, low self-efficacy, cervical cancer training and observed number of deaths) were identified.

In all, 10 respondents participated in this study. Table 1 presents the profile of these respondents in terms of their backgrounds, age and years of work experience. Their ages ranged from 29 years to 62 years, with work experience from 4 years to 38 years. Regarding their cadres, they ranged from nursing assistants as the lowest cadre to nursing sisters and nursing officers as the highest cadre. The rest of the findings is presented and grouped in themes, with the verbatim excerpts of the participants presented in italics.

**TABLE 1:** Demographic characteristics of participating primary health care nurses in Leribe district.

Participant	Nursing cadres	Age in years	Years of experience
001	Nursing Assistant	62	38
002	Nursing Sister	43	8
003	Nursing Sister	30	4
004	Nursing Sister	29	7
005	Nursing Assistant	53	15
006	Nursing Assistant	45	13
007	Nursing Assistant	39	15
008	Nursing Officer	50	19
009	Nursing Sister	53	31
010	Nursing Sister	48	23

**TABLE 2:** Summary of themes derived from the analysis of the perceptions of the Leribe primary health care nurses about cervical cancer screening.

Main themes	Sub-themes
1. Perceived susceptibility to cervical cancer	1.1. Being sexually active 1.2. Multiparity
2. Fear about perceived disease severity	2.1. Consequences of cancer
3. Perspective on the perceived benefits of cervical cancer screening	3.1. Feelings of relief 3.2. Early disease detection
4. Perceived barriers to cervical cancer screening	4.1. Fear of testing positive 4.2. Lack of results 4.3. Perceived lack of confidentiality and privacy
5. Self-efficacy in screening for cervical cancer	5.1. High self-efficacy 5.2. Low self-efficacy
6. Warning signs of action in regard to cervical cancer	6.1. Observed number of deaths 6.2. Cervical cancer training

### Theme 1: Perceived susceptibility to cervical cancer

Participants in this study acknowledged being susceptible to cervical cancer because they were sexually active and associated the risk of developing cervical cancer with multiparity.

#### Sub-theme 1.1: Being sexually active

‘I understood that I needed to be tested for cervical cancer, especially because I am sexually active and of childbearing age.’ (003, 29 years, Nursing Sister)

#### Sub-theme 1.2: Multiparity

‘I decided to get tested because I noticed that there were too many cases of cervical cancer and because I have more than two children, I thought I was at high risk of having cervical cancer.’ (001, 62 years, Nursing Assistant)

Because of this perceived susceptibility to cervical cancer, some nurses were self-motivated to get screened for it.

### Theme 2: Fear about perceived disease severity

Similarly to the preceding statements, the fear of cancer and its consequences appeared to encourage nurses to undergo screening. To underscore this issue, the participants said:

‘I feared cancer and noticed that people with cancer suffer a lot, so I did not want to suffer.’ (001, 62 years, Nursing Assistant)

#### Sub-theme 2.1: Consequences of cancer

‘Maybe it’s because we have seen so many women with cancer at certain stages, some having to undergo a hysterectomy. So, I don’t want to find myself in such situations.’ (003, 30 years, Nursing Sister)

### Theme 3: Perspective on the perceived benefits of cervical cancer screening

Some participants cited factors that encouraged them to screen for cancer, including feelings of relief after testing.

#### Sub-theme 3.1: Feelings of relief

‘I don’t know if my mind was playing tricks on me. After the workshop, I felt something like pain down here (pointing to the lower abdomen). However, after screening and receiving the results, the pain was no longer there.’ (010, 48 years, Nursing Sister)

‘Yes, because even when my time to repeat the screening has passed, I would feel like I have lower abdominal pain. So, I would regret not doing what I should have done.’ (006, 45 years, Nursing Assistant)

From these statements, it appears that the information that the nurses received and the knowledge of what needed to be done caused unusual agony (which was just psychological), which the negative results helped relieve.

#### Sub-theme 3.2: Early disease detection

Another encouraging factor was early detection of the disease. One participant stated:

‘[…*A*]lso, it is better to screen early than late when cancer has advanced.’ (001, 62 years, Nursing Assistant)

Half of the participants agreed with this sentiment. Following are some of the comments of the participants about early disease detection:

‘But when we started working and attended training, we got a clear picture that if one does not detect on time, this cancer can progress to a stage where it cannot be treated or has metastasized to other parts of the body.’ (003, 30 years, Nursing Sister)

‘I wanted to know my cancer status so that I could be treated early if I had it, and to avoid complications that would require treatment outside the country or stage in which I would be terminally ill.’ (008, 50 years, Nursing Officer)

### Theme 4: Perceived barriers to cervical cancer screening

Although some participants cited factors that encouraged them to be screened, some expressed factors that discouraged them from seeking further screening tests.

#### Sub-theme 4.1: Fear of testing positive

Perception of positive testing and their ability to handle it was a key factor that discouraged nurses from screening routinely:

‘So, after taking the test, I was stressed about my results and wondered how I would feel if they were positive. That is why I wanted them out urgently to escape the stress of waiting for the result.’ (006, 45 years, Nursing Assistant)

‘I was worried and asked myself what would happen to me if the results were positive.’ (005, 53 years, Nursing Assistant)

It is evident from these statement that nurses perceive themselves as at risk of cervical cancer.

#### Sub-theme 4.2: Lack of results

Even though nurses perceive themselves as at risk, the prolonged waiting period of the participants for their subsequent screening could be caused by the second factor, delay in receiving the results, or worse, not receiving them at all. The participants underscored this by saying:

‘[…*T*]hey performed a Pap smear whose results never returned.’ (009, 53 years, Nursing Sister)

‘Those (the results of the samples) that are taken to the referral hospital do not always come back. I think they return the results only when they are positive. When I look at the patients’ results, their turnaround time is too long.’ (001, 62 years, Nursing Assistant)

Failure to get results could be because of the inadequacy of the health system, as one participant puts it:

‘…VIA because you get the results immediately. But with a Pap smear, there is a problem; we were told that there was a backlog caused by the reagents being out of stock and so on.’ (009, 53 years, Nursing Sister)

#### Sub-theme 4.3: Perceived lack of confidentiality and privacy

Fears of positive testing and the long turnaround time of the results were not the only factors; some participants expressed concerns about the perceived lack of confidentiality and privacy of their screening partners:

‘It’s like the person conducting your screening is going to look at your private parts, and then they would talk about you … Yes, like here at work, when someone looks at you, it’s like they are saying ah, what I saw on this one, ah (laughed and said it with a condescending face).’ (005, 53 years, Nursing Assistant)

This position implies that nurses often go for screening tests in unfamiliar facilities rather than in their places of employment.

### Theme 5: Self-efficacy in screening for cervical cancer

As reported earlier, the participants’ experiences and perceptions impacted their decisions on whether or not to take subsequent screening tests.

#### Sub-theme 5.1: High self-efficacy

Those with positive experiences had high self-efficacy and were therefore confident in adopting healthy behaviour and regularly undertaking screening tests:

‘I will go to the screening without worrying about anything, knowing that anything is possible and the results can be positive or negative. When they are positive, according to the health education I received, I will just have to take care of myself. When positive, treatment is available, so I shouldn’t be concerned.’ (002, 43 years, Nursing Sister)

This statement suggests that nurses’ confidence in taking screening tests depends on the health education they receive. Therefore, nurses also need health education to make the right decision about undergoing cervical cancer screening.

#### Sub-theme 5.2: Low self-efficacy

For others, their low self-efficacy had affected their confidence in their ability to detect cervical cancer again in the future:

‘I don’t know why it has taken me this long to repeat the test, I believe it’s because this nurse of mine no longer works there. And you know, when you are not sick, you don’t feel the need to go back, especially because I was motivated by the training.’ (010, 48 years, Nursing Sister)

It appears that the low self-efficacy of this participant in reversing the screening was because of the absence of her preferred healthcare professional, whom she was familiar with and who worked in a different facility from her.

Of the two participants who indicated a preference for the HPV test, one expressed that the inaccessibility of her preferred screening method put her in low self-efficacy, as she said:

‘I am never taking the test again until the HPV test is available, then I can conduct my own screening. I don’t want to be seen naked.’ (004, 29 years, Nursing Sister)

It appears that this participant had low self-efficacy because of her past experiences of being seen naked. This reaffirms the analysis made earlier on about the perceived lack of confidentiality and privacy of nurses in their screeners, hence, the desire for screening that involves self-sampling. The preceding statements also reaffirm the analysis made earlier that health education (and/or training) plays an important role in reactivating nurses to screen. If nurses are motivated by training and health education, it is quite clear that health education can do a lot for the general public.

### Theme 6: Warning signs of action in regard to cervical cancer

In addition to the confidence of the participants in their ability to act and undergo cervical cancer screening, they also reported factors that prompt action.

#### Sub-theme 6.1: Observed number of deaths

Among factors that prompt action were the observations that the participants have made throughout the course of their careers:

‘I had never been tested and seeing that women were dying of cervical cancer, I felt I should be tested.’ (008, 50 years, Nursing Officer)

#### Sub-theme 6.2: Cervical cancer training

Another reported factor was the cervical cancer training nurses received:

‘After receiving the training, what was taught made me get screened so that when I encourage others, I also know the good and bad of it or what the consequences would be of delaying undergoing the screening.’ (007, 39 years, Nursing Assistant)

It appears that training the nurses received not only prompted action among them alone but also allowed them to have honest conversations with their clients. Therefore, it is safe to assume that frequent training can potentially positively influence cervical cancer screening among nurses and, in turn, among the general population.

## Discussion

Our study indicated the varying perceptions that encouraged or inhibited nurses from routinely screening for cervical cancer. Among those that were encouraging, the most cited was perceived susceptibility to cervical cancer among nurses, who believe they are at risk of the disease because they are sexually active and have more than two children and therefore felt the need to get tested. The results of a Turkish study, including nurses and midwives, agree with these findings. In that study, participants who had given birth once or twice were more likely than those who had never given birth to have had Pap smear tests at least once in their lifetime.^31^ These findings show that nurses are aware of the risk factors for cervical cancer, which motivates them to get screened. As a result, they may improve screening for their patients because they are aware of who is more likely to develop the disease. The perceived severity of the disease, which was caused by the observations that nurses made about the extent to which cancer patients suffered its consequences, also encouraged nurses to be screened. Therefore, the perceived severity of the disease helped nurses understand the seriousness of the negative effects of cervical cancer. As a result, they were screened for the disease to avoid these effects. In addition, nurses can enhance screening for other women to reduce the number of cancer cases and avoid encounters with cancer patients who suffer.

Furthermore, based on their perception of their own risk of cervical cancer, the nurses in this study also perceived benefits of cervical cancer screening. Thus, they thought that screening would help detect the disease early and that they would be relieved if the results were negative. These results are consistent with those of the study conducted by De Queiroz Neves et al.,^32^ in which the participants stated that they underwent the Pap smear test because it allows for early identification of cervical cancer, providing them peace of mind about their health.

In this study, what inhibited nurses from undergoing routine screening were perceived barriers to cervical cancer screening, including their ability to cope with a positive (abnormal) test result, the perceived lack of privacy and confidentiality in the screeners, especially when the screeners are their colleagues, and the delay in receiving the results. Several studies^20,33^ concur with the findings of the current study. For example, 16% of HCWs in an Indian study never had a Pap smear because of fear of bad results.^20^ Suantika et al.^20^ further emphasised that fear was believed to play a role because nurses were afraid of what would happen if they had cervical cancer and, therefore, were reluctant to know the test results. In another instance, an Australian study that involved the screening of women workers in selected PHCCs revealed that the workers found it unsettling to have their colleagues screen them in the clinic and occasionally went to other clinics to maintain privacy and confidentiality.^33^ According to these findings, nurses are more likely to avoid screening because, instead of their workplace, they would rather go to places where their screening will be done by strangers. There may be fewer recommendations for such services to other women if nurses are concerned about the delay in receiving the results. This is because nurses may be apprehensive about what they would say to these women after violating their privacy for nothing.

As previously discussed, despite the perceived barriers that discouraged nurses from undergoing routine screening, they had positive perceptions of self-efficacy and therefore persisted in undergoing screening. This is similar to a study conducted by Suantika et al.,^20^ which showed that 33 nurses who took a Pap smear test had positive perceptions of self-efficacy, but 253 nurses did not have a Pap smear performed because of negative perceptions. That study concluded that negative perceptions cause nurses to refrain from performing Pap smears and so does this study.

In addition to the high self-efficacy of the participants, certain cues encouraged them to take the screening tests. For example, the observations made by nurses regarding the number of deaths from cervical cancer. Moreover, the cervical cancer training that nurses received played a crucial role in motivating and recalling nurses to take the screening tests. These findings are consistent with a study conducted in three selected public hospitals in Ethiopia among female health professionals. According to the results of that study, respondents who had received training in cervical cancer screening were 1.6 times more likely to be screened for the disease than those who had not.^34^ These results also align with the study findings that involved women from three provinces in Turkey and found that those who received educational brochures along with one-on-one training had a higher rate of cervical cancer screening than their counterparts who were only given brochures.^35^

The older participants, that is, those over 30 years of age with more than 10 years of work experience, had more than one screening test compared to younger participants with less than 10 years of work experience. These findings are almost comparable to those of a study conducted in Nigeria with participants under 30 years of age and who had practised for 5 years or less.^17^ That study concluded that because nurses were young, they did not seem to think that they would develop precancerous or cancerous lesions and were therefore not concerned about such matters, making them less likely to use the service. Therefore, it is reasonable to assume that nurses are unlikely to offer this service to other women, particularly women of their own age. In their studies, Daglas et al.^36^ and Vishram et al.^37^ confirm this behaviour where health professionals are less likely to recommend a service if they do not personally utilise or believe that a service has no benefits; the opposite is true. Furthermore, a study conducted in Turkey^31^ found that among nurses and midwives, the frequency of Pap smear tests increased greatly with increasing age. Thus, the present study found that nurses’ decisions to get screened for cervical cancer appeared to be influenced by their age and years of work experience. This aspect needs to be explored further with a study properly designed to test this hypothesis.

### Strengths

Through the use of an exploratory qualitative design, the study explored the perceptions of PHC nurses about cervical cancer screening, as this design allowed the researcher to probe more into the area. Furthermore, this study makes an important contribution to the body of knowledge on how cervical cancer screening programmes can be enhanced in light of the perceptions of nurses that encouraged routine screening and those that discouraged nurses from routine screening.

### Limitations

Because of the design of this study, only one nursing officer participated in this study as the nursing officers at other PHCCs were unable to participate because of work restrictions. Therefore, future research should consider the availability of all nursing cadres. Since this study was unfunded and conducted by a full-time employee, the interviews were only conducted among nurses working in PHCCs located in lowland areas. Therefore, the transferability of these findings is limited. Future research with PHC nurses in remote and highland locations should be considered to explore this topic in depth.

### Recommendations

Based on the study findings, the authors recommend the following: Firstly, since most nurses were inspired to detect cervical cancer from what they learned about it, regular cervical cancer training is recommended among HCWs. Secondly, interventions to improve the turnaround time of the results should be explored, as one of the factors that deterred nurses from screening was the waiting period for the results. Thirdly, less intrusive screening modalities should be investigated to alleviate the discomfort caused by existing methods of cervical cancer screening. One of these less intrusive screening modalities, HPV testing, was only introduced in the Leribe district months after data collection. Therefore, we recommend further studies on how to improve the screening experience, as the new Guide for Human Papillomavirus Test and Management.^38^ explains that with a positive HPV test, triage tests for treatment using VIA, VILI, and Pap smear are recommended.

## Conclusion

The study shows that PHC nurses participate in cervical cancer screening. However, the problem is routine screening. Even those who had subsequent screenings had them long after the due dates. Their perceptions of cervical cancer screening are one of the factors that contribute to this and might also contribute to fewer screening recommendations and education for the general public by these health professionals who understand the importance of screening but prefer to postpone the planned screening because of these perceptions. As seen in the Results section, the most often suggested way around this obstacle is to attend routine cervical cancer training sessions, since the information they learn there encourages nurses to be screened.
